# Customized 3D-Printed Titanium Mesh Developed for an Aesthetic Zone to Regenerate a Complex Bone Defect Resulting after a Deficient Odontectomy: A Case Report

**DOI:** 10.3390/medicina58091192

**Published:** 2022-09-01

**Authors:** Gabriela Luminița Gelețu, Alexandru Burlacu, Alice Murariu, Sorin Andrian, Loredana Golovcencu, Elena-Raluca Baciu, George Maftei, Neculai Onica

**Affiliations:** 1 Department of Surgery, Faculty of Dental Medicine, University of Medicine and Pharmacy “Grigore T. Popa”, 700115 Iasi, Romania; gabriela.geletu@umfiasi.ro (G.L.G.); alice.murariu@umfiasi.ro (A.M.); loredana.golovcencu@umfiasi.ro (L.G.); 2 Department of Internal Medicine, Nephrology, Geriatrics, Faculty of Medicine, University of Medicine and Pharmacy “Grigore T. Popa”, 700115 Iasi, Romania; alexandru.burlacu@umfiasi.ro; 3 Department of Dentistry-Periodontology, Fixed Prosthesis, Faculty of Dental Medicine, University of Medicine and Pharmacy “Grigore T. Popa”, 700115 Iasi, Romania; sorin.andrian@umfiasi.ro; 4 Department of Implantology, Removable Dentures, Dental Technology, Faculty of Dental Medicine, University of Medicine and Pharmacy “Grigore T. Popa”, 700115 Iasi, Romania; 5 Private Practice, 700377 Iasi, Romania; nicuonica@yahoo.com

**Keywords:** custom-made titanium mesh, computer-aided design, computer-aided manufacturing, allograft bone, odontectomy, implants

## Abstract

*Background and Objectives*: Alveolar ridge augmentation in the complex bone defect is a popular topic in implantology. Guided bone regeneration (GBR) is one of the most commonly applied methods to reconstruct alveolar bone. The application of a membrane is the fundamental principle of GBR. There are many membrane types used in oral surgery, but the advantage of the titanium mesh is the rigidity which provides space maintenance and prevents contour collapse. The smooth surface also reduces bacterial contamination. Using computer-aided design (CAD) and computer-aided manufacturing (CAM) in dentistry allows us to obtain the perfect architecture form of the mesh, which covers and protects the bone reconstruction. *Case presentation:* We present a surgical case of a 27-year-old female patient with severe aesthetic bone atrophy after a deficient odontectomy. Based on the GBR clinical applications, the technique consists of bone reconstruction and a customized titanium mesh application. Using mesh titanium in this case presentation was a reliable alternative to perform a lateral alveolar bone augmentation and reconstruct ridge deformities before reaching an ideal implant placement. *Conclusions*: According to our case report, the customized titanium mesh could be a valuable option for guided bone regeneration in aesthetic maxillary defects.

## 1. Introduction

Many patients have insufficient alveolar bone height and width. These can be resulted from trauma, cancer, infection, or prior inadequate surgery. Bone and teeth loss determine significant aesthetic and masticatory function deficiency. The severity of bone atrophy determines the type of bone augmentation procedure. The most critical techniques for bone augmentation are guided bone regeneration (GBR), onlay and inlay grafting, maxillary sinus lift, distraction osteogenesis, alveolar ridge splitting, and free vascularized autografts [1].

The principle of GBR using barrier membranes is to exclude certain cell types such as rapidly proliferating epithelium and connective tissue, thus promoting the growth of slower-growing cells capable of forming bone. GBR is often combined with bone grafting procedures. Bone graft materials are placed in the bone defect area as scaffolds, guiding osteoblasts and osteocytes to form new bone [2,3]. A systematic review reported 95% implant survival after a horizontal or vertical GBR procedure [4]. The major components of the treatment with GBR are the membrane properties and the predictable regeneration of bone, with adequate soft-tissue reactions [5].

The improvement of membrane types acts against main disadvantages such as lack of rigidity and stability, low toughness and plasticity, and too fast resorbability. The barrier membranes commonly used clinically can be divided into resorbable and non-resorbable membranes according to their resorbability. The collagen-based membranes are the most often used naturally derived membranes for GBR due to their considerable bioresorbability and low immunogenicity. The main non-resorbable membranes are polytetrafluoroethylene (PTFE) in its expanded form ePTFE, titanium-reinforced PTFE, and titanium mesh [6]. Crucial properties of membranes are biocompatibility, cell-occlusion properties, integration by the host tissues, clinical manageability, space-making ability, and adequate mechanical and physical properties.

According to the biomaterial type, membranes can be classified into synthetic polymers, natural polymers, and metals. Synthetic polymers are non-resorbable and need a second surgery, lack rigidity, and present reduced stability. Natural polymers, such as collagen and extracellular matrices derived from bovine, porcine, and human tissues, are bioresorbable and have low immunogenicity. Resorbable collagen membranes consist of a homogenous collagenous matrix or a bilayer structure. A bilayer membrane such as BioGide (Geistlich Pharma, Wolhusen, Switzerland) has one compact layer that can prevent the infiltration of epithelial cells into the bone tissue and a second layer that stimulates integration [7]. Lack of rigidity and stability is one of the important disadvantages of synthetic polymers used for GBR [8]. The exposure of ePTFE to the oral cavity determines bacterial infection and can compromise bone augmentation and osteointegration [9].

Titanium mesh has important mechanical properties, including good plasticity through bending and shaping, and showing a high stable osteogenesis effect [10,11]. Thickness and porosity are critical factors in the utilization of titanium mesh; a 0.2 mm mesh can be suitable for most situations, offering appropriate flexibility [12].

Many studies have attempted to investigate the role of porosity of titanium mesh, and the results are controversial. The relationship between pore size and the amount of soft tissue growth is not very clearly demonstrated. Titanium has a high and persistent corrosion resistance [13]. It is observed that a thin layer of 1–2 mm thick tissue could be found upon the regenerative bone surface called “pseudo-periosteum” after bone reconstruction. This pseudo-periosteum can play a role in graft protection and infection prevention [14]. GBR with titanium mesh has strong osteogenesis predictability, and horizontal and vertical bone augmentation can be obtained with delayed or simultaneous implantation [15]. The delayed implantation strategy demonstrated an average bone augmentation of 4–5 mm in bone width and 5–7 mm in bone height [16]. Titanium mesh cannot be resorbed by the body, which means that both titanium mesh and fixation screws need to be removed through second-stage surgery, causing trauma to the patients. In contrast, the design and printing of a customized mesh that simulates the ideal reconstruction eliminate the intra-operative risk of contamination, handling, and trimming, reducing surgical time and minimizing stress for the soft tissues. This also applies to regular and rounded edges due to the 3D laser-sintering printing technology [17].

The purpose of this article is to present a surgical case with severe aesthetic bone atrophy after a deficient odontectomy.

## 2. Case Presentation

**Figure 1:**
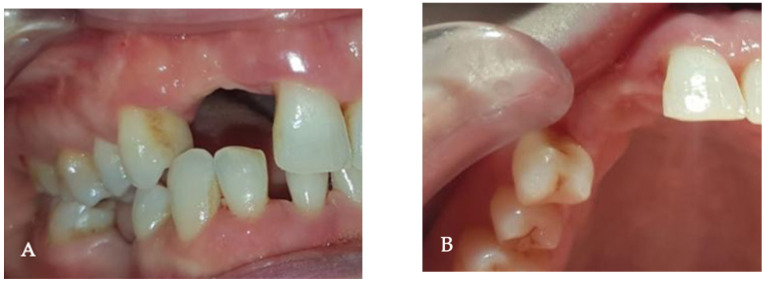
The initial intraoral aspect of the patient proposed a gingival graft before the bone augmentation: (**A**) the buccal view, (**B**) the occlusal view.

**Figure 2:**
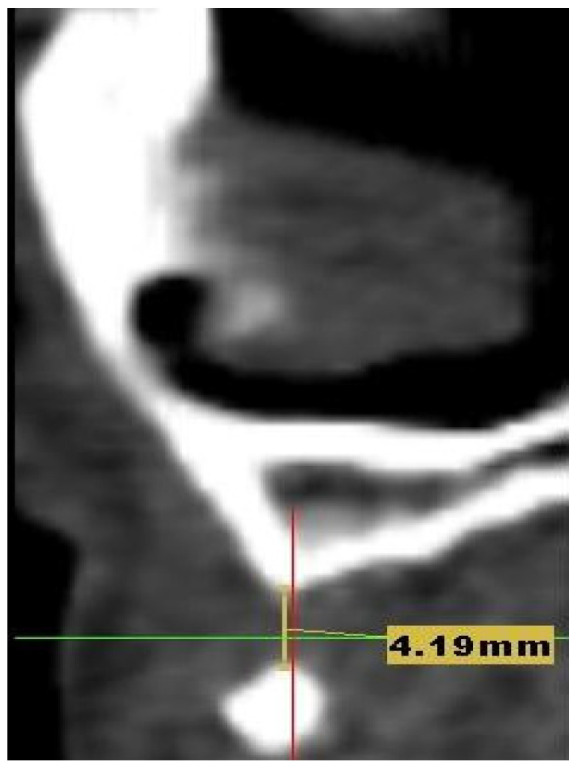
The CBCT aspect of the initial situation: the defect measures 4.19 mm in length and 4.2 mm in width of bone deficiency.

A 27-year-old woman presented to the dental clinic for aesthetic and masticatory dysfunction after a difficult odontectomy ten years ago. The provisional removable acrylic appliance (flipper) unsatisfied her and created a social disadvantage for the young woman. After a complete intraoral and extraoral examination (Figure 1A,B) and a cone-beam computerized tomography (CBCT) evaluation (Figure 2), the missing teeth 1.3, 1.2, and a complete palatal and buccal loss of bone with a marginal reconstructed alveolar bovine particles bone resulting from an earlier operation in the maxillary region were observed.

Considering the allograft bone and bovine bone as the better choice for atrophic sites and the necessity to transfer the virtual volumetric and morphologic project to the graft for the entire healing period, the GBR with pre-formed titanium mesh was taken into consideration.

**Figure 3:**
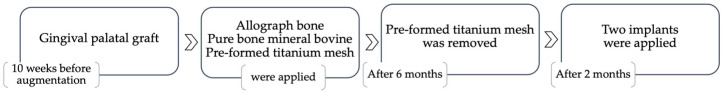
The surgical treatment plan steps.

The surgical treatment plan included several surgical procedures (Figure 3). To achieve the first goal of increasing gingival thickness, a connective tissue pedicled palatal graft was recommended. Secondary, after 10 weeks, the GBR technique planned consisted of the application of a mixed particulate bone (allograft bone and pure mineral bovine in a 1:1 ratio) and a preformed titanium mesh in the defect site resulting from the earlier odontectomy. The third intervention, scheduled for 6 months later, allowed for the removal of the mesh and evaluation of bone regeneration. Furthermore, it permitted delaying implant placement for an additional two months, allowing the usual layer of fibrous tissue underneath the titanium mesh to increase the quality of regenerated bone. Finally, it was planned for the application of two osseointegrated implants, which will permit the future prosthetic reconstruction.

Informed consent was obtained after the patient had been informed of the diagnosis, the prognosis with and without treatments, the specific therapeutic steps, advantages, procedures-specific risks, and potential adverse reactions.

**Figure 4:**
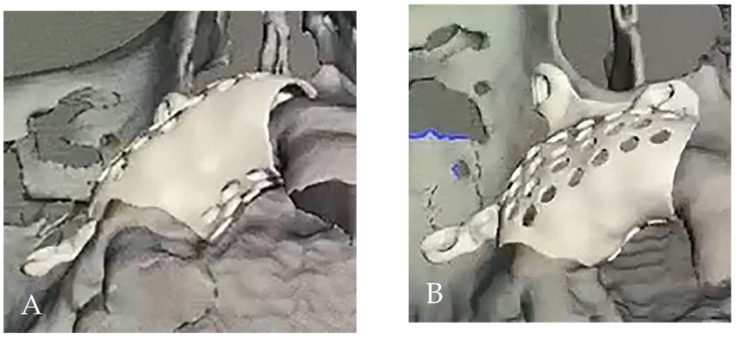
The digital design: (**A**) occlusal view, (**B**) palatal and buccal view.

**Figure 5:**
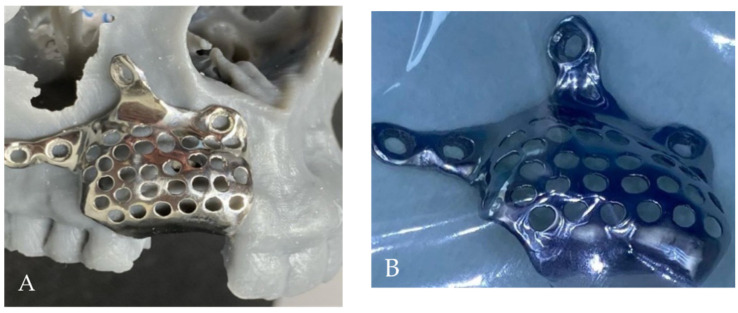
The custom-made titanium mesh: (**A**) on the 3D-printed bone model, (**B**) sterilized before surgery.

The digital model was obtained by converting the DICOM to STL-format file. Using dedicated software EXOPLAN (exocad GmbH, Darmstadt, Germany), the 3D reconstruction of the patient’s titan mesh was designed, giving the final desired form (including holes and screw holes). Then STL file of the mesh was exported to Local 3D Printing Center and printed using selective laser melting (SLM) technology (Mysint100, SISMA, Piovene Rocchette, Italy) of titanium powder (Figure 4A,B and Figure 5A,B).

**Figure 6:**
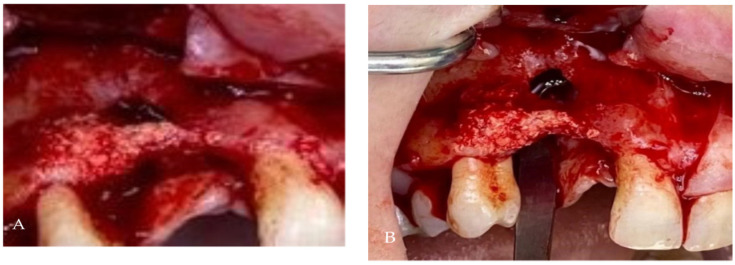
Intraoperative oral images: (**A**) the defect interested the entire width of the bone, situated at the apical level of the neighbored teeth, (**B**) a marginal of 2 mm particles augmented.

The blood tests were in the normal range. The patient received 2 g of amoxicillin and clavulanic acid one hour before surgery (Augmentin, Glaxo Wellcome Production ZI, Peyenniere, 53100 Mayenne, France) and then 1 g twice a day for 7 days. An oral and maxillofacial surgeon performed the surgical procedures. Immediately before surgery, the patient rinsed with a 0.2% solution of chlorhexidine (Curasept, Curaden Healthcare) for 1 min, and a sterile surgical drape was applied to disinfect the surgical site. Local anesthesia was induced using an articaine solution (4%) with epinephrine (1:100,000; Ubistein, 3 M ESPE). The surgery started with a mid-crestal incision into the keratinized tissue using a surgical blade No. 15c, then two vertical releasing cuts, followed by raising the full-thickness buccal and palatal flaps to expose the entire defect from the area to be augmented (Figure 6A,B).

The keratinized gingival was satisfied due to the previous gingival palatal pedicle graft; surgery was performed ten weeks earlier. The long connective tissue pedicled graft started from the mesial side of second molar and had the pedicle situated mesial. It measured 2.5 cm length and 0,5 cm width. The pedicled graft was placed on top of the bony crest in the edentulous defect and sutured using resorbable Surgicryl Monofast 5.0 (SMI AG, St.Vith, Belgium). The method’s purpose was to increase gingival thickness on the ridge defect, improving bone repair predictability, and lowering recession risk.

The buccal flap was coronally extended to assure a complete closure by gentle dissection of the periosteum.

The recipient site was then carefully cleaned and the mobile particles from the earlier surgery were removed. Then, allograft bone substitute granules (Max Graft, granules Bottis biomaterials Gmbh, Zossen, Germany) from a human donor site, with a unique cleaning process (Allotec process, Cells-Tissuebank, Austria), were selected. Due to its preserved natural bone structure and collagen content, this type of graft serves as a scaffold for natural bone regeneration and has the potential of complete remodeling into the patient’s bone. We mixed the allograft bone particles with Cerabone particles (Bottis biomaterials Gmbh, Zossen, Germany), a pure mineral bovine bone, in a ratio of 1:1. The mixture was placed facing the buccal bone plate, to fill the bone defect, until the palatal side of the wound.

**Figure 7:**
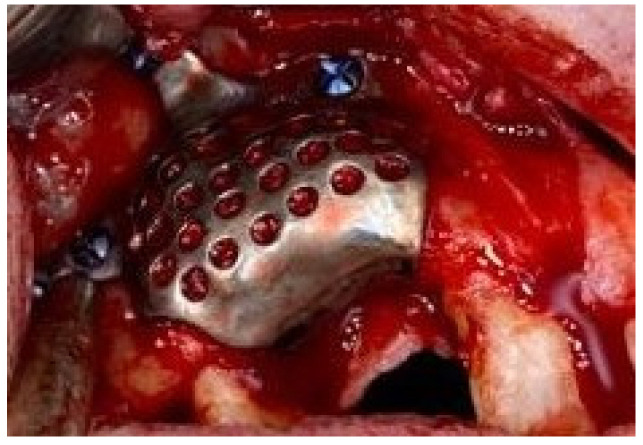
Intraoperative image: the precise application of the titanium mesh (0.5 mm thick) fixed by the screws protects the added bone-mixed particles.

The 0.5 mm pre-formed titanium mesh was precisely placed over complex alveolar bony defects and fixed with 4 self-drilling screws (Figure 7).

**Figure 8:**
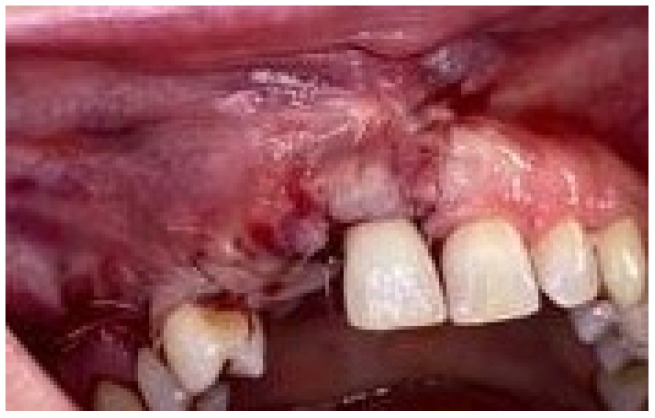
Intraoral aspect: the precise suture prevents the wound dehiscence.

The flap was carefully applied without tension and suture with a 5-0 nylon horizontal mattress technique and simple interrupted suture to achieve absolute tension-free adaptation (Figure 8). The pain and postoperative edema were treated with paracetamol and ibuprofen for three days.

In order not to jeopardize the outcome, we minimized all risk factors to prevent mesh exposure (exposed mesh can lead to bone regenerating failure). Due to this consideration, in agreement with the patient’s decision, a provisional prosthesis was no longer produced.

**Figure 9:**
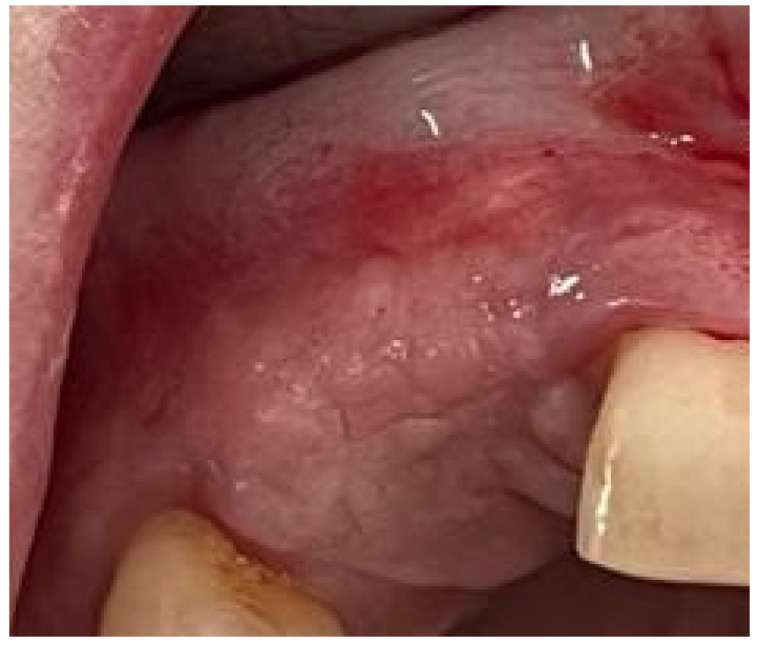
Aspect of the soft tissues (suitable for the mesh to be removed), after six months.

The healing was without inconvenience (Figure 9), with no membrane exposure, during the next 6 months.

**Figure 10:**
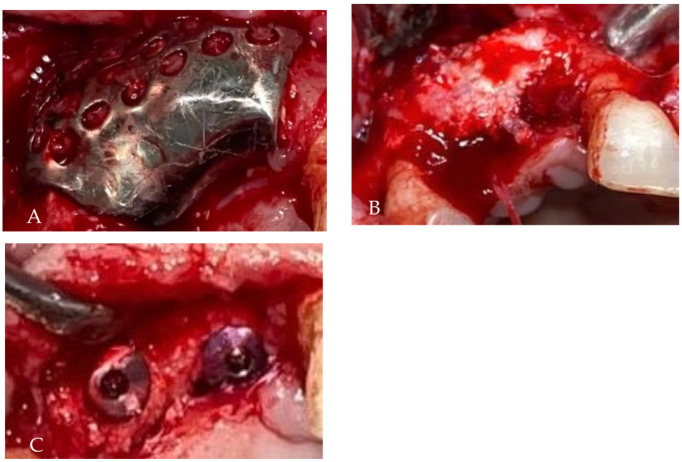
Intraoral findings: (**A**) aspect of the titanium mesh after flap elevation, (**B**) aspect of the added bone after mesh removal, (**C**) width and height of reconstructed bone were favorable and permitted the application of the implants after 2 months.

After 6 months we removed the mesh and a well-reconstructed ridge bone volume, and dense bone-like tissue were observed. After another 2 months, we reopened and new bone formation was visualized, which permitted the application of the two implants (Mis implants Technologies Ltd., Achihud, Israel) in the augmented alveolar ridge (Figure 10A–C). Both Mis implants were 3.75 mm in diameter and 11.5 mm in length. No significant complications were recorded at the recipient site.

**Figure 11:**
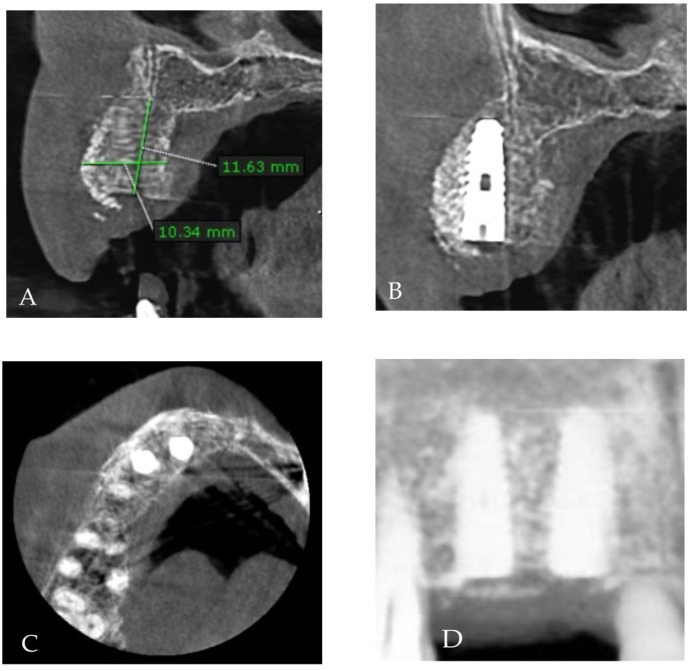
Cone-beam CT images of the implants position in the augmented bone (**A**), (**B**) sagittal view, (**C**) axial view, and (**D**) coronal view.

After three months, a CBCT (Figure 11A–D) evaluated the aspect of the osseointegrated implants and the aspect of the surrounding bone. An 11.63 mm length and a 10.34 mm width of augmented bone were obtained.

During the patient’s recovery, no provisional removable acrylic denture (flipper) was applied.

**Figure 12:**
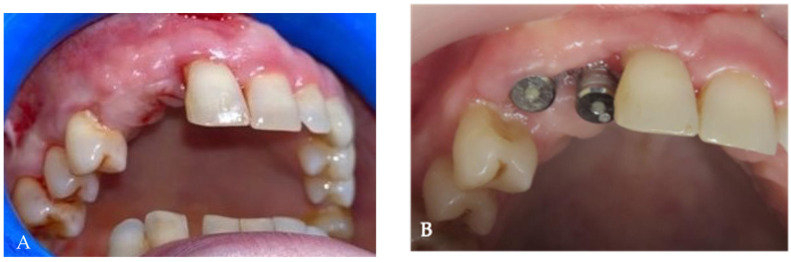
Intraoral images: (**A**) the aspect of gingival tissue before application of the healing abutments, (**B**) the aspect of gingival tissue after application of the healing abutments.

The healing period was without any complications (Figure 12A,B). Abutment connection was carried out four months after implant placement. Clinical parameters and probing depth after prosthetic reconstruction demonstrated the presence of a healthy peri-implant mucosa.

## 3. Discussion

Insufficient bone volume for a dental implant in the maxillary anterior segment is a constant challenge in oral surgery. Several techniques have been suggested to reconstruct deficient alveolar ridges and facilitate dental implant placement. These techniques include bone splitting osteotomy, distraction osteogenesis, inlay, onlay bone grafting, sinus lift, and GBR. GBR is an excellent alternative that increases bone volume using a subperiosteal barrier.

Titanium mesh is a functional non-resorbable membrane to treat alveolar bone deficiencies. It has good biocompatibility and space maintenance and can easily fit the complicated morphological bony defect. It is demonstrated that the exposure rate of titanium mesh is lower than that of polytetrafluoroethylene membranes [18].

Currently, there are various clinical procedures for bone augmentation with titanium mesh, which can be roughly divided into titanium mesh bone augmentation in simultaneous implantation, titanium mesh bone augmentation with delayed implantation, and GBR with titanium mesh in combination with other bone augmentation methods [19,20]. In our case, the titanium mesh augmentation with delayed implantation was decided.

The main disadvantage of the titanium mesh is the cost and the necessity of a second surgery to eliminate it (which needs the same total mucoperiosteum flap for its application). We remarked on the favorable augmented bone during this second surgery, removed the mesh, and inserted the dental implants.

This case report shows that a customized titanium mesh can have many advantages. Easy to manipulate, it also reduces the operation time, has minimal risk of dehiscence, and has an excellent volume of reconstructed bone. After six months, the clinical and CBCT aspect presents a sufficient bone volume offering the possibility of implant application. The osseointegration and the final aspect were favorable, permitting the application of the aesthetic bridge.

## 4. Conclusions

We have performed a maxillary reconstruction after a deficient odontectomy, using allograft and bovine particulated bone and a 3D custom-made titanium mesh (fabricated from a 3D model generated from CT data). The results were excellent, the reconstructed bone permitting the application of two implants. With the limitations of a single case report, we presented a successful maxillary reconstruction using a custom-made titanium mesh. Further research is necessary in the promising field of digitally-designed devices.

## Data Availability

Not applicable.
